# A vacuolar iron-transporter homologue acts as a detoxifier in *Plasmodium*

**DOI:** 10.1038/ncomms10403

**Published:** 2016-01-20

**Authors:** Ksenija Slavic, Sanjeev Krishna, Aparajita Lahree, Guillaume Bouyer, Kirsten K. Hanson, Iset Vera, Jon K. Pittman, Henry M. Staines, Maria M. Mota

**Affiliations:** 1 Instituto de Medicina Molecular, Faculdade de Medicina Universidade de Lisboa, 1649-028 Lisbon, Portugal; 2 Institute for Infection & Immunity, St. George's, University of London, London SW17 0RE, UK; 3 Sorbonne Universités, UPMC Univ Paris 6, CNRS, UMR 8227, Comparative Physiology of Erythrocytes, Station Biologique de Roscoff, CS 90074, 29688 Roscoff, France; 4 Faculty of Life Sciences, University of Manchester, Manchester M13 9PT, UK; 5 Present address: University of Texas at San Antonio, Department of Biology and STCEID, San Antonio, Texas 78249, USA

## Abstract

Iron is an essential micronutrient but is also highly toxic. In yeast and plant cells, a key detoxifying mechanism involves iron sequestration into intracellular storage compartments, mediated by members of the vacuolar iron-transporter (VIT) family of proteins. Here we study the VIT homologue from the malaria parasites *Plasmodium falciparum* (PfVIT) and *Plasmodium berghei* (PbVIT). PfVIT-mediated iron transport in a yeast heterologous expression system is saturable (*K*_m_∼14.7 μM), and selective for Fe^2+^ over other divalent cations. PbVIT-deficient *P. berghei* lines (*Pbvit*^−^) show a reduction in parasite load in both liver and blood stages of infection in mice. Moreover, *Pbvit*^−^ parasites have higher levels of labile iron in blood stages and are more sensitive to increased iron levels in liver stages, when compared with wild-type parasites. Our data are consistent with *Plasmodium* VITs playing a major role in iron detoxification and, thus, normal development of malaria parasites in their mammalian host.

## Abstract (web summary)

Iron is an essential nutrient but, in high concentrations, it is also toxic to cells. Here, Slavic *et al*. identify an iron transporter in malaria parasites that plays a major role in iron detoxification and is required for the parasite's normal development.

## Graphical abstract

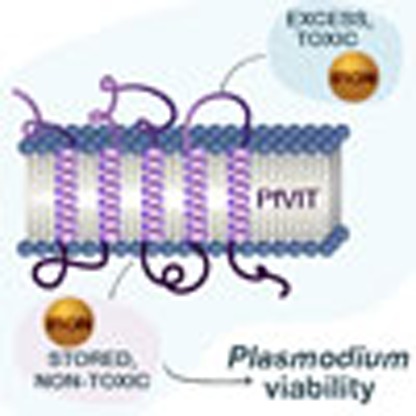

Malaria imposes a massive global health burden, with a current WHO estimate of around 600,000 deaths annually^1^, although this figure could rise sharply if treatment failures associated with artemisinin-combination therapies becomes widespread. Perturbation of iron homeostasis is an attractive strategy to target malaria parasites as *Plasmodium*, like all cells, requires iron to survive^2^,^3^. Indeed, iron is an essential micronutrient, necessary for fundamental cellular processes such as ATP and DNA synthesis. While iron's redox-active nature makes it essential for many catalytic processes, it also underlies its toxicity when present at high concentrations in the cytosol or other sites. As such, all organisms have evolved a wide range of strategies to acquire necessary iron and to detoxify any excesses.

Iron enters cells through the activity of membrane transporters or receptor-mediated endocytosis. While most of the intracellular iron gets safely stored either in complex with the iron-storage protein ferritin (for example, plant and mammalian cells) or inside organelles (for example, yeast and plant cells), a small amount of total cellular iron is present as a metabolically available pool in the cytoplasm, termed the labile iron pool (LIP). The LIP consists of iron loosely bound to small negatively charged molecules and proteins^4^ and provides iron for cellular processes including haem and Fe–S cluster biosyntheses^5^.

Appropriate storage of any excess of iron, which is not used metabolically, is essential to prevent cellular toxicity due to engagement of iron in Fenton-type chemistry in the presence of oxygen and production of potentially damaging reactive oxygen species^6^. Ferritin represents the most common and ancient mechanism of iron storage and homeostasis in nature, as it is found in most bacteria, archaea, plants and animals, but not in yeast^7^. In the absence of ferritin, the yeast vacuole serves as the main iron-storage/sequestration organelle. In response to demands, iron moves to and from the yeast vacuole through the activity of iron transporters in the yeast vacuolar membrane; CCC1 (Ca^2+^-sensitive cross complementer 1) is proposed to import iron, while a complex constituted by Smf3p and Fet5p-Fth1p exports iron^8^,^9^,^10^. Thus, vacuolar sequestration by CCC1 in yeast is likely to be the primary mechanism for detoxification of excess iron in this organism. In addition to ferritin found in plastids, plants also have several homologues of CCC1, named vacuolar iron transporters (VITs), which are likely to transport not only iron but also other divalent cations such as manganese and zinc into the vacuole for storage and detoxification^11^,^12^,^13^.

*Plasmodium* parasites do not contain a homologue of ferritin or any other known iron-storage protein^14^. However, *Plasmodium* spp. genomes contain one orthologue of plant VIT and yeast CCC1 proteins. Thus, we hypothesized that mechanisms regulating intracellular iron storage in malaria parasites may resemble those of yeast and plants. To test this hypothesis, we assessed the ability of *Plasmodium falciparum* VIT, PfVIT, to transport iron and sought to reveal the role of this protein in the establishment and course of a malaria infection by generating *Plasmodium berghei* parasites deficient in VIT (*Pbvit^−^*). We now show that *Plasmodium* VITs, expressed throughout the parasite's life cycle, transport iron and play a major role in iron detoxification in the parasite.

## Results

### *Plasmodium* genomes contain a VIT homologue

**Figure 1:**
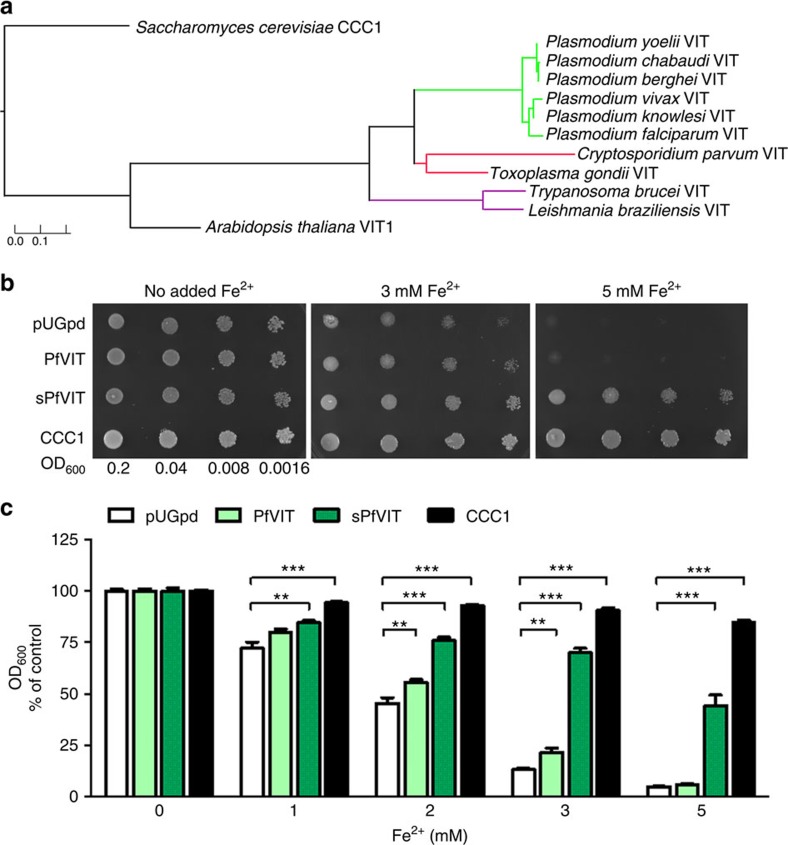
Iron tolerance of ΔCCC1 yeast conferred by the functional expression of PfVIT. (**a**) Phylogenetic analyses of *Plasmodium* VIT proteins compared with other *Apicomplexa* and *Kinetoplastida* parasites and unrelated model organisms. The phylogenetic tree was generated using Phylogeny.fr^59^ with yeast CCC1 sequence used as an out-group and visualized with TreeGraph 2 (ref. 60). The scale bar for the branch lengths is shown. (**b**) Complementation of the ΔCCC1 yeast mutant growth phenotype by expression of PfVIT. The ΔCCC1 strain (lacking vacuolar iron uptake) was transfected with empty vector pUGpd or vector expressing full-length PfVIT, N-terminally truncated PfVIT (sPfVIT) or CCC1. Transfected strains were diluted (as indicated by OD_600_ values), spotted onto SD agar plates lacking histidine and supplemented with: no additional Fe^2+^, 3 mM Fe^2+^ and 5 mM Fe^2+^ and grown at 30 °C for 48 h. Fe^2+^ was provided as ammonium FeSO_4_ in the presence of 1 mM ascorbic acid. (**c**) The ΔCCC1 transfectants described in **b** were inoculated at a cell density of 0.01 OD_600_ in SD medium in the presence of indicated Fe^2+^ concentrations (provided as ammonium FeSO_4_ in the presence of 1 mM ascorbic acid) and grown with shaking at 30 °C for 20 h. Yeast cell density was determined by absorbance measurements at 600 nm and for each strain shown the OD_600_ values are normalized to those obtained in the absence of additional Fe^2+^ in the medium (controls, 0 mM Fe^2+^). Data are shown as the means±s.e.m. of a pool of three independent experiments, each performed in triplicate. The data were analysed using the unpaired, two-tailed Student's *t*-test; **P<0.01, ***P<0.001.

Bioinformatic analysis shows that all *Plasmodium* spp. genomes encode one gene with homology to yeast CCC1 and plant VIT proteins—PF3D7_1223700 in *P. falciparum* and PBANKA_143860 in *P. berghei*. PfVIT shares 28% amino-acid sequence identity with *Arabidopsis thaliana* VIT, AtVIT1, and 19% with *Saccharomyces cerevisiae* CCC1 (ClustalW2 multiple protein alignment). Like VIT homologues in other organisms, both PfVIT and PbVIT are predicted to have five transmembrane domains (Supplementary Fig. 1). PfVIT has four phospho-acceptor sites at amino-acid positions 21S, 122S, 140S and 150T, as reported previously^15^,^16^. VITs are highly conserved across *Plasmodium* spp., with open reading frames (ORF) split between two exons. Orthologues in the species infecting humans (*P. falciparum*, *P. vivax* and *P. knowlesi*) form a separate phylogenetic branch from the orthologues of rodent malaria species (*P. yoelii*, *P. chabaudi* and *P. berghei*; Fig. 1a). VIT homologues are also present in other human pathogens of the *Apicomplexa* phylum, such as *Toxoplasma* and *Cryptosporidium.* Furthermore, kinetoplastid parasites *Trypanosoma* and *Leishmania*, causing sleeping sickness and leishmaniasis, respectively, also encode homologues of VIT proteins (Fig. 1a).

### PfVIT complements a yeast strain lacking CCC1

*S. cerevisiae* CCC1 most likely transports Fe^2+^ and Mn^2+^ into the vacuole, based on the effect of *CCC1* deletion and overexpression on vacuolar accumulation of both of these ions^8^. Mutants lacking the encoding gene for CCC1 (Δ*CCC1*) are susceptible to iron toxicity in the presence of high extracellular iron concentrations but can be rescued by expression of homologous plant VITs, such as AtVIT1 (ref. 11), *Tulipa gesneriana* VIT1 (ref. 12) and *Oryza sativa* VIT1 and VIT2 (ref. 13). To assess whether *Plasmodium* VITs could rescue Δ*CCC1* susceptibility to iron toxicity, a yeast expression vector (pUGpd) containing codon-optimized full-length *pfvit* (*pfvit* pUGpd; encoding 273 amino acids) and 5′-truncated *pfvit* (*spfvit*-pUGpd; encoding 236 amino acids and containing all 5 predicted transmembrane domains, Supplementary Fig. 1) were transformed into *S. cerevisiae* Δ*CCC1*. The latter was used because truncated transporters in heterologous expression systems often demonstrate increased activity, for example, because of autoinhibitory effects^17^. Parallel transformation with empty vector (pUGpd) and vector containing CCC1 (CCC1-pUGpd) were used as negative and positive controls, respectively. Having confirmed that the appropriate sized gene sequences were present in Δ*CCC1::*PfVIT, Δ*CCC1::*sPfVIT and Δ*CCC1::*pUGpd yeast strains, western blot analysis of whole cell protein extract demonstrated that full-length PfVIT heterologous protein expression was very low compared with that of the truncated version, with the latter being easily detectable in vacuolar membrane fractions (Supplementary Fig. 1). Subsequently, yeast growth assays on solid (qualitative) and in liquid (quantitative) media were performed (Fig. 1b,c). The results show that very low-level expression of PfVIT provided moderate but significant rescue of the ΔCCC1 phenotype at 2 and 3 mM Fe^2+^ (*P*<0.01, Student's *t*-test; Fig. 1c). However, highly expressed sPfVIT significantly and strongly rescued the ΔCCC1 phenotype at all tested Fe^2+^ concentrations (*P*<0.01; Fig. 1c), although not to the degree measured in the CCC1-expressing yeast positive control (which may be the result of relatively low protein expression of heterologous sPfVIT sequence compared with that of CCC1, or else it may represent true differences in transport properties between these two transporters, such as in substrate affinity and transporter capacity). Additionally, Zn^2+^ tolerance was also tested by expression of sPfVIT in a Δ*zrc1* yeast strain, which is sensitive to increased Zn^2+^ concentrations due to the lack of a vacuolar Zn^2+^ importer^18^,^19^. Expression of sPfVIT did not rescue Δ*zrc1* from sensitivity to high external Zn^2+^ concentration (Supplementary Fig. 2).

These data show that PfVIT, particularly when truncated, can be expressed in *S. cerevisiae* and can complement the lack of CCC1, implying that PfVIT acts as an iron transporter.

### PfVIT mediates Fe^2+^ uptake

**Figure 2:**
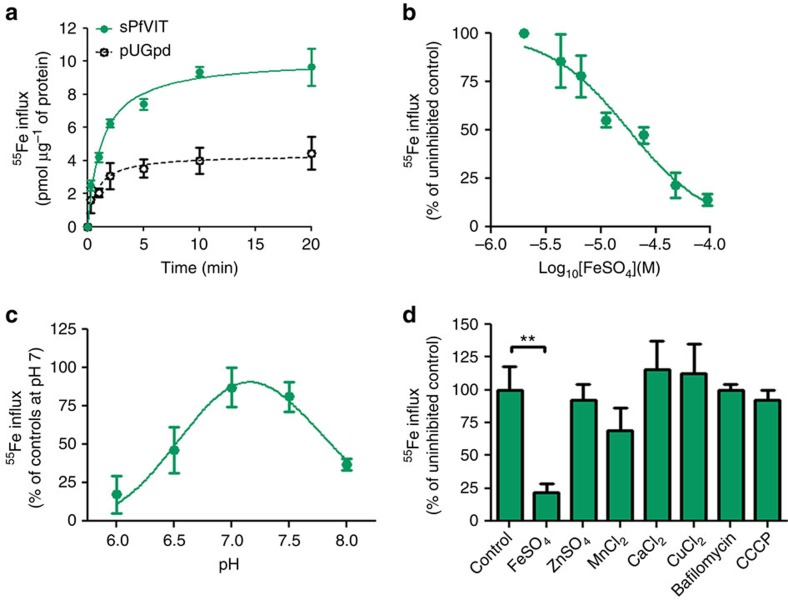
Characterization of iron transport by PfVIT. ^55^Fe influx was measured into vacuole-enriched vesicles isolated from ΔCCC1 yeast transfected with empty or sPfVIT-expressing pUGpd vectors. (**a**) ^55^Fe uptake over time, measured at pH 7. (**b**) Inhibition of sPfVIT-mediated ^55^Fe^2+^ influx (defined as the influx in Δ*CCC1::*sPfVIT isolated vacuoles minus that measured in Δ*CCC1::*pUGpd vacuoles) by ‘cold' unlabelled iron, measured over 1 min at pH 7. Shown is sPfVIT-mediated ^55^Fe^2+^ influx in the presence of increasing concentrations of FeSO_4_ normalized to the condition without FeSO_4,_ to allow comparison between independent vacuolar preparations. (**c**) pH dependence of sPfVIT-mediated ^55^Fe^2+^ influx, measured over 1 min, normalized to that obtained at pH7. (**d**) Inhibition of sPfVIT-mediated ^55^Fe^2+^ influx by 100 μM divalent metals, 1 μM bafilomycin A1 and 20 μM CCCP, measured over 1 min at pH 7, normalized to uninhibited control sPfVIT-mediated ^55^Fe^2+^ influx (**P<0.01; one-way analysis of variance, Dunnett's Multiple Comparison Test). In all panels, data are shown as means±s.e.m. of 6–8 influx measurements performed at room temperature.

We next decided to test the ability of sPfVIT to transport Fe^2+^. We measured uptake of ^55^Fe^2+^ into vacuolar vesicles isolated from transfected Δ*CCC1::*sPfVIT yeast. ^55^Fe^2+^ uptake was over twofold higher in these vacuoles, when compared with vector-only control vacuole preparations (Δ*CCC1::*pUGpd) that were made at the same time, reaching 80% of maximum uptake after 5 min of incubation at 20 °C (Fig. 2a). The same fold increase in ^55^Fe^2+^ uptake was also observed between paired sPfVIT and control vacuolar vesicles made on different days, even though uptake relative to total protein levels varied considerably (compare uptake presented in Fig. 2a with that presented in Supplementary Fig. 3a). Using the same paired vacuolar preparations used to generate the data presented in Fig. 2a, linear-phase ^55^Fe^2+^ influx in Δ*CCC1::*sPfVIT isolated vacuoles measured over 1 min was 4.2±0.3 pmol min^−1^ μg^−1^ protein (mean±s.e.m.; *n*=9), which was significantly higher than that measured in the same vacuoles placed on ice (1.9±0.3 pmol min^−1^ μg^−1^ protein; mean±s.e.m.; *n*=9; *P*=0.0003; two-tailed, Student's *t*-test) and in control vacuoles (2.1±0.3 pmol min^−1^ μg^−1^ protein; mean±s.e.m.; *n*=8; *P*=0.0002; two-tailed, Student's *t*-test). sPfVIT-mediated ^55^Fe^2+^ influx (defined as the influx in Δ*CCC1::*sPfVIT vacuoles minus that measured in Δ*CCC1::*pUGpd vacuoles and normalized to account for the variability noted above when averaging data generated from more than one paired vacuolar preparation) was inhibited by unlabelled Fe^2+^ in a concentration-dependent manner (Fig. 2b) and was found to be pH-sensitive, with an optimum between pH 6.5 and 7.5 (Fig. 2c). In competition assays with divalent metals at 50-fold higher concentrations (that is, at 100 μM), no other metal inhibited sPfVIT-mediated ^55^Fe^2+^ uptake (Fig. 2d). This included Zn^2+^, and thus is in agreement with the Δ*zrc1* rescue assay described above. These data demonstrate that PfVIT is selective for Fe^2+^. Given the vacuolar localization of the majority of VIT, they have been proposed to act as Fe^2+^/H^+^ exchangers. Thus it is interesting that the observed transport via sPfVIT occurred without the need to acidify the yeast vacuoles by the addition of ATP, which activates endogenous V-type H^+^ pumps. To support this finding, we further demonstrated that Fe^2+^ transport via sPfVIT was insensitive to the V-type H^+^ pump inhibitor bafilomycin A1 or to the proton ionophore CCCP (Fig. 2d). Furthermore, to address the possibility that the transport measurements via sPfVIT are an artefact of Fe^2+^ vesicle surface binding, several additional experiments were performed (Supplementary Fig. 3b). Adding the divalent cation chelator EDTA to the wash solution used during transport experiments (see Methods section) had no effect on ^55^Fe^2+^ uptake, while lysing vacuolar vesicle preparations by either three freeze-thaw cycles before experimentation or addition of the detergent Triton X-100 at the start of a 1-min transport measurement significantly reduced ^55^Fe^2+^ uptake. Finally, lysis of vesicles using 0.1 M HCl added directly following a 1-min transport experiment significantly reduced accumulated ^55^Fe^2+^. These results are consistent with ^55^Fe^2+^ accumulating within vesicles rather than adsorbing/binding to vesicles.

### PbVIT is expressed throughout the parasite's life cycle

**Figure 3:**
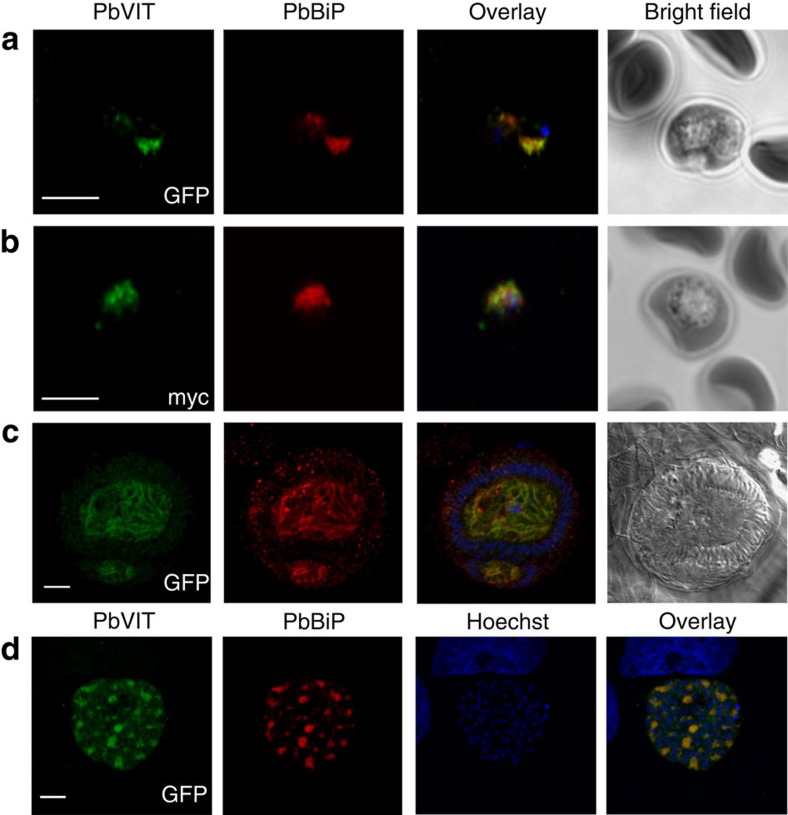
Expression of PbVIT in blood-, mosquito- and liver-stage parasites. Indirect immunofluorescence assay of PbVIT-GFP and PbVIT-myc *P. berghei.* (**a**) Blood-stage PbVIT-GFP *P. berghei* (shown is a trophozoite stage parasite). (**b**) Blood-stage PbVIT-myc *P. berghei* (shown is a trophozoite stage parasite). (**c**) A mosquito midgut oocyst (17 days post infection). (**d**) A liver-stage EEF, 48 h post infection of Huh7 the hepatoma cell line with PbVIT-GFP sporozoites. Stainings were performed using antibodies against GFP, myc (green) and PbBiP (ER marker, red), and Hoechst nuclei stain (blue).

Published transcriptomic data show that *Pfvit* is expressed in *P. falciparum* blood-stage parasites with its abundance increasing as the parasite progresses from early ring to mature trophozoite stage^20^,^21^,^22^. We next generated a C-terminal green fluorescent protein (GFP) or myc fusion of PbVIT in *P. berghei* rodent parasites, using a single crossover transfection strategy (Supplementary Fig. 4). This allows the study of VIT expression and localization throughout the parasite's life cycle. Confocal analysis after immunostaining with anti-GFP or anti-myc antibodies showed PbVIT expression in asexual blood stages, mosquito and liver stages of infection (Fig. 3 and Supplementary Fig. 6). In blood-stage parasites, both tagged versions of PbVIT mainly co-localized with PbBiP to the parasite's endoplasmic reticulum (Fig. 3a,b and Supplementary Fig. 5). We did not observe PbVIT-GFP signal in regions around hemozoin crystals in blood-stage parasites. In oocysts and liver-stage parasites PbVIT-GFP also co-localized with PbBiP (Fig. 3c,d). Altogether, these data strongly support the idea that PbVIT is expressed mainly in the parasite ER throughout the parasite's life cycle.

### *Pbvit^−^* parasites yield reduced liver and blood infections

**Figure 4:**
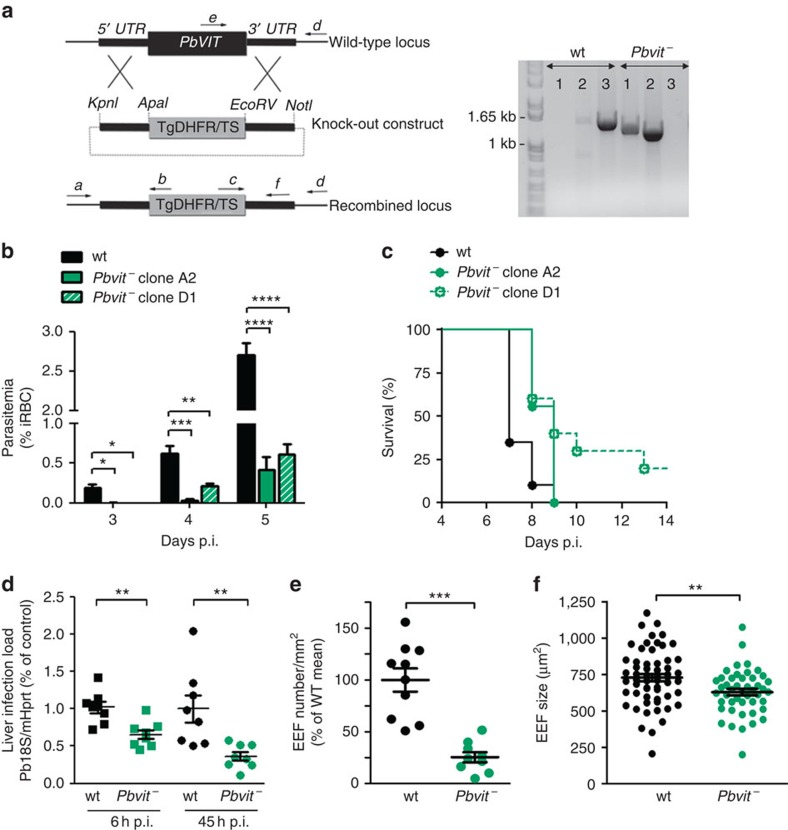
*Pbvit* knockout affects blood- and liver- stage growth of *P. berghei.* (**a**) Double crossover strategy for *pbvit* knockout and genotyping of the *Pbvit−* transgenic clonal line by PCR. Lane 1, detection of knockout construct integration at the 5′ end (primers a+b, 1.38 kb); lane 2, knockout construct integration detection at the 3′ end (primers c+d, 1.23 kb); lane 3, wt *pbvit* locus (primers e+d, 1.45 kb). (**b**) Parasitemia of C57Bl/6J mice following infection (*i.v.*) with 10^4^ wt *P. berghei* or *Pbvit^−^* iRBCs, determined by counting of iRBC in Giemsa-stained blood smears (*N*=10 for wt *P. berghei*-infected mice and *N*=5 for mice infected with *Pbvit^−^* A2 or D1). (**c**) Survival of C57Bl/6 J mice infected *i.v.* with 10^4^ wt or *Pbvit^−^* iRBC (*N*=20 for wt *P. berghei*-infected mice and *N*=10 for mice infected with *Pbvit^−^* A2 or D1). Median survival was 7 and 9 days for wt and *Pbvit^−^*, respectively, *P*<0.01, log-rank Mantel–Cox test. (**d**) Parasite liver load 6 and 45 h after *i.v.* injection of wt or *Pbvit^−^* sporozoites, assessed by reverse transcription PCR measurement of parasite 18s RNA expression, normalized to mouse hypoxanthine-guanine phosphoribosyltransferase, shown are fold expressions relative to the average of controls–wt *P. berghei* (shown is the pool of two independent experiments). (**e**) Number of EEFs per mm^2^ of livers 45 h after infection with 50,000 wt and *Pbvit^−^* sporozoites. Each point represents an average number of EEFs per mm^2^ per mouse liver by counting the number of EEFs in 5–8 slices per liver (shown is a pool of two independent experiments, in total 795 wt EEFs and 210 *Pbvit^−^* EEFs were counted). (**f**) Size of liver EEFs determined by measuring the PbUIS4 surrounded area by ImageJ in confocal images of liver sections. The mean±s.e.m. size of wt and *Pbvit^−^* EEFs was 728±25 (*N*=58) and 629±23 (*N*=46) μm^2^, respectively. In **b**,**d**,**e** and **f** error bars represent s.e.m. and the asterisks denote significant differences using the two-tailed, unpaired Student's *t*-test: *P<0.05; **P <0.01 and ***P<0.001.

Our data have revealed a novel *Plasmodium* iron transporter whose homologues in yeast and plants act as iron detoxifiers. We therefore asked whether *Plasmodium* VITs play a similar role during the parasite's life cycle. To answer this question, we generated a *P. berghei* parasite line deficient in the *Pbvit* gene (*Pbvit^−^*), using a double crossover transfection strategy (Fig. 4a). *Pbvit^−^* parasites were cloned to obtain isogenic mutant lines, which were used for all further analyses. Successful knockout of *pbvit* demonstrates a non-essential role for this transporter during asexual *P. berghei* blood stages. However, when controlled experimental infections were performed, parasitemias in *Pbvit^−^* (two independent clones A2 and D1) infected mice were significantly reduced, when compared with those in mice infected with wild-type (wt) parasites (*P*<0.05, Student's *t*-test; Fig. 4b). Furthermore, C57Bl/6 J mice infected with *Pbvit^−^* parasites (10^4^ infected RBC *i.v.*) survived significantly longer compared with mice infected with wt parasites (*P<*0.01, log-rank Mantel–Cox test; Fig. 4c).

Notably, while *Pbvit^−^* did not show any defect during transmission to or development within *Anopheles stephensi* mosquitoes (Supplementary Fig. 7), a significant reduction in parasite liver load was detected after *Pbvit^−^* sporozoite infection. Indeed, to investigate if *Plasmodium* VITs play a role during the first obligatory stage of *Plasmodium* infection in the mammalian host, *Pbvit^−^* or wt *P. berghei* sporozoites obtained from *A. stephensi* mosquitoes were *i.v.* injected into C57Bl/6 J mice and parasite liver load was measured 6 and 45 h later. The data show a moderate but significant reduction in infection with *Pbvit^−^* (clone A2) sporozoites 6 h after injection (37±10% lower, when compared with wt *P. berghei* infection; *P*<0.01, Student's *t*-test; Fig. 4d). Furthermore, 45 h after infection the parasite liver load was reduced by 64±19% for *Pbvit^−^* clone A2 (*P*<0.01; Fig. 4d) and by 53±19% for *Pbvit^−^* clone D1 (*P*<0.05; Supplementary Fig. 7), when compared with wt *P. berghei* infection. Analysis of infected mouse liver sections, by microscopy, show that the observed reduction in parasite liver load was due to a lower number of infected hepatocytes (Fig. 4e), as well as moderate reduction in size of developing *Pbvit^−^* exoerythrocytic parasite forms—EEFs (Fig. 4f). The combined effect of *Pbvit^−^* deficiency on both liver and blood stages was also observed following an entire course of infection initiated with 500 *Pbvit^−^* and wt sporozoites (Supplementary Fig. 7).

### Pbvit provides an iron detoxification mechanism

**Figure 5:**
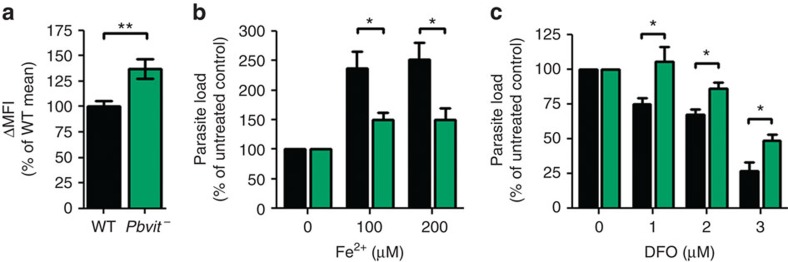
PbVIT functions in iron detoxification by reducing the LIP. (**a**) The LIP of *P. berghei* wt and *Pbvit^−^* iRBCs analysed by flow cytometry. ΔMFI was determined by evaluating the change in mean fluorescence intensity of PhenGreen-loaded iRBCs (SYTO 61-positive subset), after incubation with 100 μM DFO (ΔMFI=MFI_DFO treated_−MFI_DFO untreated_). For each independent experiment, the MFI of *Pbvit^−^* iRBCs was normalized to the mean MFI of wt-iRBCs. Shown is a pool of four independent experiments (*N*=14), **P=0.0028 (unpaired, two-tailed Student's *t*-test; wt mean±s.e.m.=100±6, *Pbvit^−^* mean±s.e.m.=137.1±10). (**b**) Liver-stage parasite load in HepG2 cells 45 h post infection with *P. berghei* wt and *Pbvit^−^* sporozoites in the absence or presence of FeSO_4_ and ascorbic acid in the growth medium, normalized to internal untreated control. Liver stage parasite load was determined by reverse transcription PCR quantification of parasite 18s expression normalized to human hypoxanthine-guanine phosphoribosyltransferase expression (shown is a pool of five independent experiments). (**c**) Parasite load in HepG2 cells 45 h post infection in the absence or presence of DFO added to the growth medium, determined as in **b** and normalized to internal untreated control (shown is a pool of five independent experiments). In **b** and **c** the asterisks denote significant differences using the two-tailed, unpaired Student's *t*-test: *P<0.05.

In yeast and plants, VIT proteins maintain the homeostasis of iron and some other divalent metals by their sequestration from the cytoplasm into organelles. Thus, to investigate the physiological role of PbVIT and test our hypothesis that it detoxifies excess iron by sequestration, we compared the LIP in *Pbvit^−^* and wt *P. berghei* parasites. To that end, iRBCs containing either *Pbvit^−^* or wt parasites were stained with the iron-sensitive fluorescent probe, PhenGreen, and analysed by flow cytometry (Supplementary Fig. 8). The results show that the LIP of *Pbvit^−^* iRBCs was significantly higher than that of wt *P. berghei* iRBCs (37±11% increase; *P*<0.01, Student's *t*-test; Fig. 5a).

Since the percentage of sporozoite-infected hepatoma cells *in vitro* is extremely low, the PhenGreen flow cytometry approach is not appropriate to study the role of PbVIT during liver-stage infection. We therefore employed an alternative strategy by comparing sensitivities of *Pbvit^−^* and wt liver-stage parasites to iron depletion *in vitro*, by adding an iron chelator (deferoxamine, DFO), and iron complementation, by adding Fe^2+^ (FeSO_4_ in the presence of 1 mM ascorbic acid). We determined the DFO EC_50_ for wt parasites developing in HepG2 cells to be 2.7 μM (95% CI 2.5–3 μM; Supplementary Fig. 9). Therefore, DFO concentrations of 1, 2 and 3 μM were used for further experiments comparing *Pbvit^−^* and wt parasites. For iron supplementation, concentrations of up to 250 μM FeSO_4_ in the growth medium did not have deleterious effects on the growth of wt parasites and HepG2 cell viability (Supplementary Fig. 9) and, thus, concentrations of 100 and 200 μM FeSO_4_ were used in further experiments. While under control conditions parasite liver load of *Pbvit^−^* parasites was not different from that of wt parasites (*P*=0.16, one sample *t-*test), *Pbvit^−^* parasite load was lower compared with that of wt parasites in conditions of excess iron (Fe^2+^; Fig. 5b), consistent with a role for PbVIT in iron detoxification. Conversely, *Pbvit^−^* parasites better tolerated chelation of iron by DFO (Fig. 5c) and established higher parasites loads compared with wt parasites in the presence of DFO in the growth medium. Taken together, we show that *Pbvit^−^* parasites harbour increased LIPs and are more sensitive to environmental fluctuations in iron levels, leading to either growth defects under excess iron conditions or growth rescue under iron chelating conditions. Therefore, these data strongly support a key role for PbVIT in cellular iron detoxification in both blood and liver stages of infection.

## Discussion

Regulation of iron is essential for cell survival and should also, therefore, be critical during the entire *Plasmodium* life cycle^23^,^24^,^25^,^26^. Iron withdrawal by the use of iron chelators has been explored as an antimalarial approach for decades^27^ and novel compounds with improved pharmacokinetic properties are being investigated^23^,^24^. During infection, growth of malaria parasites is influenced by the host iron status both in liver^25^,^28^ and blood^29^. *P. falciparum* growth is reduced in iron-deficient erythrocytes while iron supplementation eliminates this growth attenuation^30^. However, the influence of iron deficiency and iron supplementation on malaria disease progression is complex and remains a controversial topic because of conflicting epidemiological and laboratory data^31^.

How malaria parasites import, export and store iron is not currently understood in detail. Recently a *P. berghei* metal transporter, ZIPCO, was described as important for the parasite's development in the liver^26^. While no direct transport studies were performed to characterize the transport properties of ZIPCO; iron and zinc supplementation and depletion experiments suggested it plays a role in uptake of iron and zinc across the plasma membrane^26^. Mechanisms used by the parasite to store and detoxify excess iron remain unknown. Asexual erythrocyte stage parasites face high demands for maintaining iron homeostasis as they digest iron-containing haemoglobin and the LIP increases with their maturation from ring to schizont forms^32^. VIT family members have been described in plants and yeast^8^,^11^,^12^,^13^,^33^,^34^ and, more recently, in the human pathogen *Trypanosoma brucei*^35^ as important iron regulatory mechanisms. Our study is the first to characterize VIT homologues in *Plasmodium* and the first to confirm the iron transport properties of any VIT homologue.

We show here that expression of PfVIT restores transport of Fe^2+^ into the vacuoles of yeast cells lacking CCC1—the yeast VIT homologue, thereby rescuing the growth defective phenotype of this strain in conditions of increased extracellular Fe^2+^. Interestingly, the rescue of the ΔCCC1 growth phenotype was far greater when the N-terminal tail, before the first predicted transmembrane region, was removed (resulting in sPfVIT). This is similar to the study of Ca^2+^/H^+^ exchangers (CAXs), including the *P. falciparum* CAX, where the N-terminal tail has autoinhibitory properties^17^,^36^. It is worth noting that the N-terminus of PfVIT contains a phospho-acceptor site, suggesting regulation by phosphorylation. However, our data suggest that removal of the N-terminus improves greatly the expression and subsequent delivery of sPfVIT to the yeast vacuole and, thus, improving its function.

While numerous VITs have been studied, the functional characteristics of Fe^2+^ transport via VITs have yet to be demonstrated. Here we have measured ^55^Fe^2+^ uptake into isolated yeast vacuoles expressing sPfVIT (confirmed by western blot). sPfVIT-mediated ^55^Fe^2+^ uptake was consistent with transport via a Fe^2+^-specific carrier protein, with an estimated *K*_m_ value of approximately 15 μM, that was relatively insensitive to pH between 6.5 and 7.5 (note the physiological range of the parasite cytosol is pH 7.1–7.3 ref. 37). The *K*_m_ value would suggest that PfVIT is a relatively low affinity/high capacity transport pathway, given that LIP measurements in malaria parasite iRBCs are 0.2–2 μM (ref. 38). Most VITs have been proposed to be Fe^2+^/H^+^ exchangers, given their localization and proposed role (see below), with Fe^2+^ being accumulated into acidic vacuoles. Yet our data were produced without the need to acidify the yeast vacuoles by the addition of ATP, which activates endogenous V-type H^+^-ATPases (and was unaffected by either a specific inhibitor of this ATPase or a H^+^ ionophore). Whether, PfVIT is an exchanger or facilitative transporter and whether it is a typical member of this family of transporters remains to be established.

In plants and yeast, VITs are usually localized to the membrane of their acidic vacuoles transporting excess iron from the cytoplasm into the vacuole. Consistent with this, a *T. brucei* VIT homologue has recently been shown to localize to acidocalcisomes^35^. We initially hypothesized that *Plasmodium* VIT would be expressed in the membrane of the parasite's food vacuole, where haemoglobin digestion and subsequent iron-containing haem detoxification occurs. However, while food vacuoles are only observed in asexual blood stages of development, analysis of published transcriptome and proteome studies suggested expression of *Plasmodium* VITs throughout the entire parasite's life cycle^15^,^39^,^40^,^41^. Generation of a C-terminal GFP or myc fusion of PbVIT in *P. berghei* allowed us to confirm complete life cycle expression. Additionally, colocalization studies with selected parasite organelles' markers imply PbVIT localization in the parasite ER, in both liver and blood stages of infection. While future studies are necessary to confirm that non-tagged versions of VIT are indeed in the ER, two plant transporters with demonstrated functional homology to CCC1 have been shown to localize to the ER, more specifically to ER bodies, where they act to maintain transition metal homeostasis^42^. Thus, our study paves the way to explore further the role of the ER in iron detoxification.

As VIT and CCC1 homologues in plants and yeast, respectively, are transporters that remove excess iron from the cytoplasm and prevent iron toxicity, we therefore hypothesized that *Plasmodium* VIT performs a similar function during the parasite's life cycle. Consistent with this, *Pbvit^−^* parasites have higher LIPs in blood stages and are more sensitive to increased iron levels in liver stages, when compared with wt parasites. Most importantly, *Pbvit^−^* parasites show a reduction in parasite load in both liver and blood stages of infection, also consistent with *Plasmodium* VIT playing a major role in iron detoxification and highlighting the necessity for iron detoxification if malaria parasites are to remain viable in the mammalian host.

Notably, while our data show that PbVIT is important for both liver and blood stages of infection, it is not essential during the entire *P. berghei* life cycle. This may reflect a redundancy of function with other compensatory mechanisms preventing iron toxicity. In *S. cerevisiae* for example lack of vacuolar iron uptake by CCC1 knockout is compensated by increased iron import into mitochondria through MRS3 and MRS4 transporters^43^,^44^. Additional protection mechanisms against high iron toxicity in *S. cerevisiae* involve induction of iron–sulfur cluster-binding proteins, such as TYW1 (ref. 45), an enzyme that participates in the synthesis of wybutosine. Thus, in addition to sequestration of iron into organelles, yeast cells avoid iron toxicity by consumption of free cytosolic iron through the formation of protein-bound iron–sulfur clusters. *Plasmodium* orthologues of mitochondrial iron importers *mrs3* and *mrs4* remain to be identified, as does the putative role of the *Plasmodium* orthologue of TYW1 and other iron–sulfur cluster containing proteins in mediating protection against high iron toxicity in malaria parasites.

In conclusion, our data provides new insights into how iron is regulated within *Plasmodium* parasites. This knowledge is highly relevant to better understand parasite biology and with regard to malaria treatment and drug resistance. Artemisinins interact with iron *in vitro* and iron chelators antagonize artemisinins in these models^46^,^47^, although acute experiments in uncomplicated malaria have not confirmed antagonism *in vivo*^48^. However, the effects of altered iron concentrations within the erythrocyte and the parasite itself remain to be systematically studied with regard to the efficacy of artemisinin therapies. As such, of further interest is to investigate altered drug sensitivity especially to quinolones or artemisinins in parasites lacking VIT.

## Methods

### Yeast expression of PfVIT

The ΔCCC1 yeast strain and CCC1-expression plasmid^8^ were kindly provided by Jerry Kaplan's laboratory, University of Utah, USA. A codon-optimised version of *pfvit* ORF (GenScript, USA Inc.) was used for yeast expression (Supplementary Fig. 1). The expression plasmid was constructed by subcloning the *BamH*I-*Xba*I codon-optimised *pfvit* fragment into the expression vector pUGpd, containing the Ura selectable marker, yeast centromere sequence and autonomously replicating sequence, which confers mitotic and meiotic stability^49^. The N-truncated fragment of codon-optimized *pfvit* ORF was amplified using primers sPfVITf and sPfVITr (Supplementary Table 1) and also subcloned into the pUGpd vector. PfVIT and sPfVIT expressing pUGpd constructs as well as CCC1-expression plasmid and empty pUGpd plasmid were transformed into the Δ*CCC1* yeast strain (lacking the VIT CCC1)^8^. sPfVIT-pUGpd and empty pUGpd were also transformed into the Δ*zrc1* yeast strain (lacking a vacuolar zinc transporter, ZRC1)^18^. Yeast strains were transformed using a previously described Li–acetate method^50^ and selected on SD medium lacking uracil.

### Preparation of vacuolar membrane vesicles from yeast

Yeast vacuolar vesicles for ^55^Fe transport assays were isolated from Δ*CCC1*::PUG and Δ*CCC1*::sPVIT-transfected strains according to a previously described protocol^51^, with the following modifications: ultracentrifugation steps for initial pelleting of microsomal membranes was at 120,000*g*, the subsequent sucrose gradient was at 150,000*g* and the final pelleting of vacuoles was at 150,000*g* and were all performed for 45 min at 4 °C. The final vacuolar fraction was resuspended in 5 mM Tris-MES, pH 7.6, 0.3 M sorbitol, 1 mM dithiothreitol, 1 mM PMSF and 1x protease inhibitor (cOmplete, EDTA-free, Sigma-Aldrich) and frozen in liquid nitrogen until use. Vacuolar preparations were always prepared as paired from Δ*CCC1*::PUG and Δ*CCC1*::sPVIT on the same day, and paired preparations were used in the transport experiments.

### ^55^Fe transport assays

For ^55^Fe uptake assays, vacuole vesicles at protein concentration of 5–10 μg per reaction were prepared in reaction solution containing 0.3 M sorbitol, 5 mM 3-(N-Morpholino)propanesulfonic acid (MOPS) (pH 7), 25 mM KCl, 1 mM dithiothreitol, 0.2 mM Na-azide and 1 mM ascorbic acid. Reactions were performed at pH 7, except for experiment to determine the effect of pH. Uptakes were started by the addition of ^55^Fe at a final concentration of 6 μCi ml^−1^, corresponding to 2 μM Fe. Uptake reactions were performed at room temperature (∼20 °C) and on ice. At the times indicated, aliquots (100 μl) of the reaction mix were removed and filtered through premoistened 0.45 μm pore-size cellulose acetate GS type filters (Millipore) and washed three times with 1 ml ice-cold washing solution containing 0.3 M sorbitol, 5 mM Tris-MES (pH 7.5), 25 mM KCl, 100 μM FeSO_4_. For EDTA wash condition used to test the unspecific binding of ^55^Fe, 100 μM EDTA was added to the wash solution. The filters were air-dried and radioactivity was determined by liquid scintillation counting. ^55^Fe influx was normalized to the protein content of the each vacuolar sample used in the experiment.

### Western blot analysis

Microsomal and vacuolar preps from Δ*CCC1*::pUGpd, Δ*CCC1*::PVIT and Δ*CCC1*::sPVIT yeast were subjected to SDS–PAGE (7 μg of protein was loaded for each) and electrotransferred to a nitrocellulose membrane. For a loading control, membranes were stained with Ponceau S. After washing, membranes were blocked in 5% skimmed milk and 0.1% Tween 20 in PBS for 3 h at room temperature. Membranes were then probed with 1:500 diluted anti-PfVIT antibody (affinity purified peptide polyclonal goat antibody, antigen sequence CGLIVTNEDKNE, from Genscript) overnight at 4 °C and then incubated with 1:5,000 diluted HRP-conjugated secondary antibody. The signal was detected with Luminata Crescendo Western HRP substrate (Millipore) and imaged with the ChemiDoc XRS+ system.

### Animal work

C57BL/6J and BALB/c wt mice were purchased from the Charles River Breeding Laboratories and were housed in the facilities of the Instituto de Medicina Molecular in Lisbon. All *in vivo* protocols were approved by the internal animal care committee of the Instituto de Medicina Molecular and were performed according to national and European regulations.

### *P. berghei* transfection and culture

Transfection experiments were performed on *P. berghei* ANKA strain 2.34 parasites according to a described protocol^52^. The *pbvit* knockout vector was constructed for a double crossover homologous recombination, as previously described^53^. Primer sequences used to amplify 5′ and 3′ untranslated regions are given in Supplementary Table 1. The final knockout construct was digested with *Kpn*I and *Not*I to release the fragment for transfection. The pyrimethamine-resistant parasite population containing the correct genomic integration of the *pbvit* knockout construct was cloned by injecting one parasite per mouse (BALB/c male mice, 6–8 weeks of age). To generate the *pbvit-gfp* transfection construct (single crossover homologous recombination), a 0.8 kb region of the *pbvit* was amplified without the stop codon and inserted in frame and upstream of the *gfp* sequence in the transfection plasmid containing the human *dhfr* cassette and conveying resistance to pyrimethamine. Similarly, *Pbvit-myc* transfection construct was generated by inserting the same 0.8 kb region of the *pbvit* in frame and upstream of the *myc* sequence in the transfection plasmid containing the human *dhfr* cassette. Transfected *P. berghei* parasites were selected with pyrimethamine selection pressure according to a described protocol^52^.

For liver-stage experiments, *A. stephensi* mosquitoes (produced by Instituto de Medicina Molecular insectary) were fed on BALB/c male mice (6–8 weeks of age) infected with wt, *Pbvit^−^* and *PbVIT-GFP P. berghei.* For collection of salivary gland sporozoites, infected mosquitoes were dissected on the 21st day post infection.

### Genotype analysis of *P. berghei* transfectants

PCR analysis performed on genomic DNA isolated from transgenic *P. berghei* was used to inspect if the transfection constructs integrated into the correct loci in pyrimethamine-resistant parasites. Sequences of primers used for genotyping are provided in Supplementary Table 1.

### Culturing and infection of hepatoma cells

HepG2 and Huh7 hepatoma cells (ATCC, USA) were cultured in supplemented Dulbecco's modified Eagle's medium or RPMI 1640, respectively, and maintained in a 5% CO_2_ humidified incubator at 37 °C. For determination of the parasite infection load *in vitro*, 50,000 HepG2 cells were plated per well of a 24-well culture plate and 24 h later infected with 30,000 wt or *Pbvit^−^* sporozoites per well. Post infection, hepatoma cells were cultured in the presence of 0.3% Fungizone (added to the culture medium). After 45 h of incubation under standard culture conditions in the presence or absence of DFO (1–3 μM) or FeSO_4_ (100–200 μM) added to the culture medium, infected cells were collected for RNA extraction and parasite load was analysed by Real-time PCR (Supplementary Table 1).

### Analysis of the PbVIT-GFP localization

BALB/c male mice (6–8 weeks of age) infected with PbVIT-GFP *P. berghei* were bled and the suspension of infected blood in RPMI medium was passed through a CF-11 cellulose column to remove leukocytes. After three washes in PBS, blood stages of PbVIT-GFP *P. berghei* were immunostained according to a previously described protocol^54^. For liver-stage localization experiments, HepG2 or Huh7 hepatoma cells were seeded on imaging coverslips in a 24-well culture plate and infected with *PbVIT-GFP P. berghei* sporozoites. Coverslips were fixed at indicated time points post infection in 4% paraformaldehyde/PBS solution for 15 min at room temperature. After three washes in PBS, coverslips were incubated for 45 min in a permeabilization/blocking solution containing 0.1% Triton X-100 and 2% bovine serum albumin in PBS. Coverslips were then incubated for a minimum of 2 h at room temperature in the primary antibody solution diluted accordingly in the permeabilization/blocking solution. Following three washes in PBS, coverslips were then stained with a mixture of appropriate secondary antibodies, diluted 1:500 in the permeabilization/blocking solution. All stainings for indirect immunofluorescence assay (IFA) presented in Fig. 3 were performed using a monoclonal mouse anti-GFP antibody (Abcam; 1:500). Additional, control stainings presented in Supplementary Fig. 4 were performed using a rabbit polyclonal anti-GFP antibody (Abcam; 1:500). For colocalization experiments, rabbit anti-PbBiP was used at a 1:600 dilution and goat anti-PbUIS4 diluted 1:1,000. The secondary antibodies used were: donkey anti-mouse conjugated to Alexa Fluor 488, donkey anti-rabbit conjugated to Alexa Fluor 549 and donkey anti-goat conjugated to Alexa Fluor 660. All images of PbVIT-GFP-expressing parasites were captured with a Zeiss LSM 710 confocal point-scanning microscope. Hoechst 33342 was used for nuclear staining.

The PbBiP antibody (rabbit, polyclonal) was designed to recognize a highly conserved C-terminal region of PBANKA_081890 (GANTPPPGDEDVDS) based on previously widely used *P. falciparum* BiP antibody^55^, which also cross-reacted with *P. berghei*^56^.

### Immunohistochemical staining of liver sections

Livers isolated from infected mice were fixed with 4% paraformaldehyde at room temperature for 2 h. The fixed liver lobes were cut into 50-μm-thick sections using the Vibratome VT 1000S (Leica). Following blocking in 2% bovine serum albumin and 0.3% Triton X-100 at 4 °C overnight, liver sections were stained with goat anti-*P. berghei* UIS4 (1:1,000) (ref. 57) and mouse anti-*P.* berghei HSP70 (1:1,000) (ref. 58). The secondary antibodies used for detection were: Alexa Fluor 555 donkey anti-goat antibody and donkey anti-mouse conjugated to Alexa Fluor 488 (all 1:500). Cell nuclei were stained with diamidino-2-phenylindole. Stained liver sections were mounted on microscope slides with Fluoromount-G (SouthernBiotech). Images were acquired on a LSM 710 confocal point-scanning microscope (Zeiss).

### Determination of the LIP of iRBCs

The LIP of RBCs infected with wt and *Pbvit^−^* parasites was determined by flow cytometry using the PhenGreen fluorescent iron probe. The staining protocol before flow cytometry measurement was based on a recently published method for *P. falciparum*^32^ with the following modification: infected BALB/c male mice (6–8 weeks of age) with 1.5–2.8% parasitemia were bled and the RBCs incubated in culture overnight to enrich the culture for mature parasite stages. RBCs were washed in PBS and stained with PhenGreen for 45 min (10 μM probe in serum-free RPMI 1640 medium). Following two washes in PBS, cells were incubated for 1 h in standard culture conditions with 0.5 μM Syto61 DNA stain, in the presence or absence of 100 μM DFO or 100 μM FeSO_4_+1 mM ascorbic acid. After washing, stained cells were analysed on a FACSCalibur. The geometric mean of PhenGreen fluorescence for the FL1-H, FL4-H subset (Supplementary Fig. 7) was determined for all samples. The amount of labile iron was estimated for each sample as that relative to the DFO condition (ΔMFI).

## Additional information

**How to cite this article**: Slavic, K. *et al*. A vacuolar iron-transporter homologue acts as a detoxifier in Plasmodium. *Nat. Commun.* 7:10403 doi: 10.1038/ncomms10403 (2016).

## Supplementary Material

Supplementary Information Supplementary Figures 1-9, Supplementary Table 1 and Supplementary References
