# Supplementary material for: A vacuolar iron-transporter homologue acts as a detoxifier in Plasmodium
Source: Nat Commun. 2016 Jan 20;7:10403. doi: 10.1038/ncomms10403 (PMC4735874; doi:10.1038/ncomms10403)
Supplement: Supplementary Information — Supplementary Figures 1-9, Supplementary Table 1 and Supplementary References [file ncomms10403-s1.pdf]

Supplementary Figures

Supplementary Figure 1

a

```
PfVIT      MVSKKTIEARKAYYNEDVVLSKEAHDFYHNLDKHGENHNLDKDNLKTIIFGSLDGIITIF
PbVIT      MGKQKIIDARKAYYEGDIEKSKEIHSHYHNLDKHAEHHSLDKDHLKTIIFGSLDGIITIF
           * .:* *:******: *:  *** *..*******.*.*.****.****************

PfVIT      AIVSGCVGAKITPTQVIIIGIGNLFANAISMGFSEYTSSTAQRDFMLAEKKREEWEIENC
PbVIT      AIVSGCVGANITPAQVIIIGVGNLFANAISMGFSEYTSSTAQIDFMLAERQREEWEIENC
           *********:***:******:********************* ******::*********

PfVIT      PSEEKQEMIDIYMNKYKFDSEDARNLVEITFRNKNFFLEHMMSEELGLIVTNEDKNECLK
PbVIT      PTEEKQEMIDIYINKYKFDSKDAKNLVEITFRNKHFFLEHMMSEELGLILTNEDKSEAFK
           *:**********:*******:**:**********.**************:*****.*.:*

PfVIT      KGIIMFLSFAVFGIIPLSAYVAYTVFFGYTDYTTSFLVVFISTLTTLFILGLFKSQFTNQ
PbVIT      KGILMFLSFCFFGMIPLFSYVLYNLFFSAENYTSSFAVVFISTLITLFILGLFKSQFTTQ
           ***:*****..**:*** :** *.:**.  :**:** ******* *************.*

PfVIT      KPITCALYMVLNGMIAGMVPFLLGVVLKNNISE
PbVIT      KPIVCALSMVLNGSIAGMLPFLFGVLLKTNSGD
           ***.*** ***** ****:***:**:**.* .:
```

b

```
ATGGTCTCTAAAAAGACTATTGAAGCAAGAAAAGCCTACTATAACGAAGATGTTGTATTGTCCAAGGAAGCCCA
CGATTTCTATCACAATTTGGATAAACATGGTGAAAACCACAACTTGGATAAGGACAACTTAAAGACTATCATCT
TCGGTTCTTTAGACGGTATCATAACAATCTTCGCTATCGTATCAGGTTGTGTTGGTGCAAAGATTACACCAACC
CAAGTTATAATCATAGGTATTGGTAATTTGTTCGCTAACGCAATATCCATGGGTTTTAGTGAATATACTTCTTC
AACAGCACAAAGAGATTTCATGTTGGCCGAAAAGAAAAGAGAAGAATGGGAAATAGAAAATTGCCCTTCCGAAG
AAAAGCAAGAAATGATCGACATCTATATGAATAAGTACAAGTTCGATTCTGAAGACGCCAGAAACTTGGTTGAA
ATCACCTTTAGAAATAAGAACTTTTTCTTAGAACATATGATGTCTGAAGAATTGGGTTTGATCGTTACAAACGA
AGATAAGAACGAATGTTTGAAAAAGGGTATCATCATGTTCTTATCCTTCGCAGTCTTTGGTATTATACCATTGA
GTGCCTATGTCGCTTACACCGTATTTTTCGGTTACACTGATTACACTACATCTTTCTTAGTTGTCTTTATCTCA
ACTTTGACCACTTTGTTTATTTTGGGTTTATTCAAATCACAATTCACTAACCAAAAGCCTATAACCTGCGCTTT
GTACATGGTTTTAAACGGTATGATCGCTGGTATGGTTCCTTTCTTGTTGGGTGTCGTCTTGAAAAATAATATCT
CCGAATAA
```

c

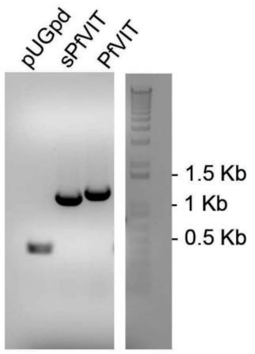

d

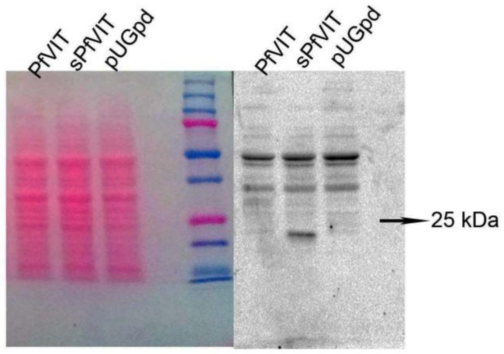

e

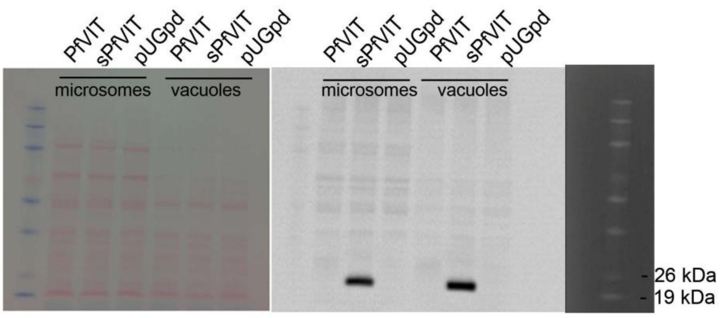

Supplementary Figure 1 | Expression of PfVIT in ΔCCC1 yeast mutant. (**a**) Amino acid sequence analysis of PfVIT and PbVIT. PfVIT and PbVIT share 78.4% of amino acid sequence identity (Clustal Omega multiple sequence alignment). Transmembrane domains, highlighted in grey, were predicted with TMHMM Server v.2.0. B. Previously reported phosphorylation sites are highlighted in yellow [1,2]. Amino acids shown in green were truncated in sPfVIT generated for functional expression in yeast strain ΔCCC1. (**b**) Codon-optimised sequence of PfVIT used for expression in yeast. (**c**) Genomic DNA analysis of ΔCCC1::pUGpd, ΔCCC1::sPfVIT and ΔCCC1::PfVIT yeast using PCR was performed to confirm the presence of correct plasmid. Primers used anneal in the 5' and 3' region of the pUGpd plasmid and amplify fragments of 0.4 Kb, 1.1 Kb and 1.2Kb for empty pUGpd plasmid, sPfVIT and PfVIT-pUGpd, respectively. (**d**) Western blot analyses of whole cell protein extract from ΔCCC1::PfVIT, ΔCCC1::sPfVIT and ΔCCC1::pUGpd. 50 µg of protein was loaded for all samples and subjected to SDS-PAGE; after the transfer of protein to nitrocellulose membrane, staining with Ponceu S confirms equality of protein loading between samples (left image). After blocking, the membrane was probed with 1:500 diluted anti-PfVIT antibody and 1:5000 diluted HRP-conjugated secondary antibody, and imaged with ChemiDoc XRS+ system (right image). (**e**) Western blot analyses of microsomal and vacuolar preparations from ΔCCC1::PfVIT (full length), ΔCCC1::sPfVIT (truncated) and ΔCCC1::pUGpd (empty vector control). 15 µg of protein was loaded for all samples and subjected to SDS-PAGE; Left, Ponceu S staining of membranes after the transfer of protein; right, Western blot using anti-PfVIT antibody as above.

Supplementary Figure 2

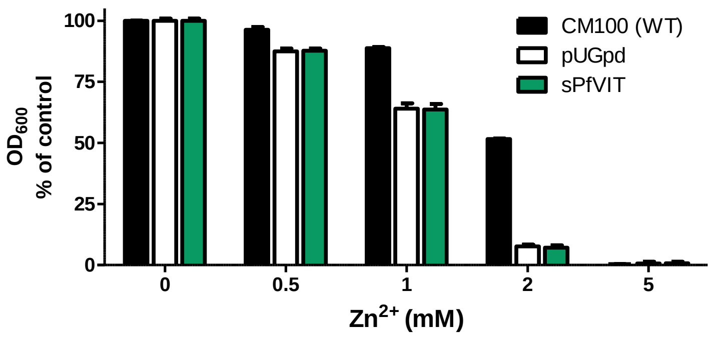

Supplementary Figure 2 | Expression of sPfVIT in Δ*zrc1* yeast mutant. Δ*zrc1* (lacking vacuolar zinc uptake) was transfected with empty vector- pUGpd or pUGpd containing N-terminal truncated PfVIT (sPfVIT). Δ*zrc1*::PUGpd, Δ*zrc1*::sPfVIT and wild type parental strain, CM100, were inoculated at a cell density of 0.01 A_{600} in in YPD medium containing indicated concentrations of $ZnSO_4$. Cell density was determined after growth at 30 °C for 20h. Shown are OD_{600} values normalised to controls - 0 mM Zn^{2+} (means ± S.E.M of 2 independent experiments, each performed with duplicates).

Supplementary Figure 3

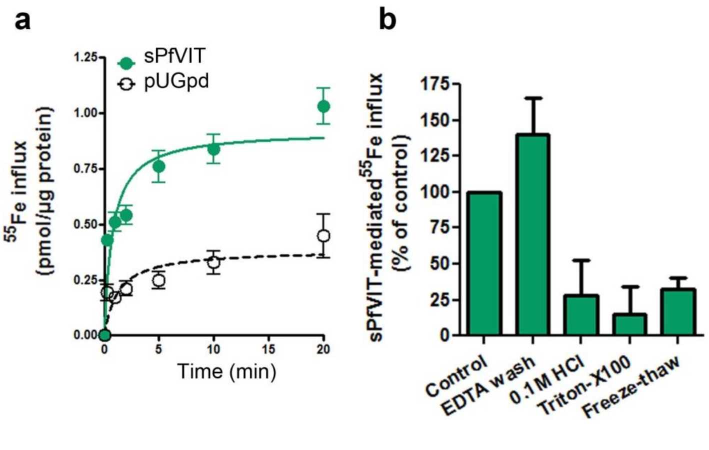

Supplementary Figure 3| Iron transport by PfVIT. (**a**) ^{55}Fe influx into sPfVIT and empty vector control vacuoles over time, measured at pH 7. (**b**) sPfVIT-mediated $^{55}Fe^{2+}$ influx (defined as the influx in sPfVIT vacuoles minus that measured in pUGpd vacuoles) measured over 1 min at pH 7, in the condition when 100 µM EDTA was added

to the washing solution, when vacuoles were lysed by addition of 0.1M HCl after the 1 min uptake, in the presence of 0.25% Triton X-100 in the reaction and when uptake was performed with vacuoles previously subjected to 3 freeze-thaw cycles. Shown data are result of 3 independent experiments (means ± S.E.M) each performed with duplicates, and normalised to the control condition.

Supplementary Figure 4

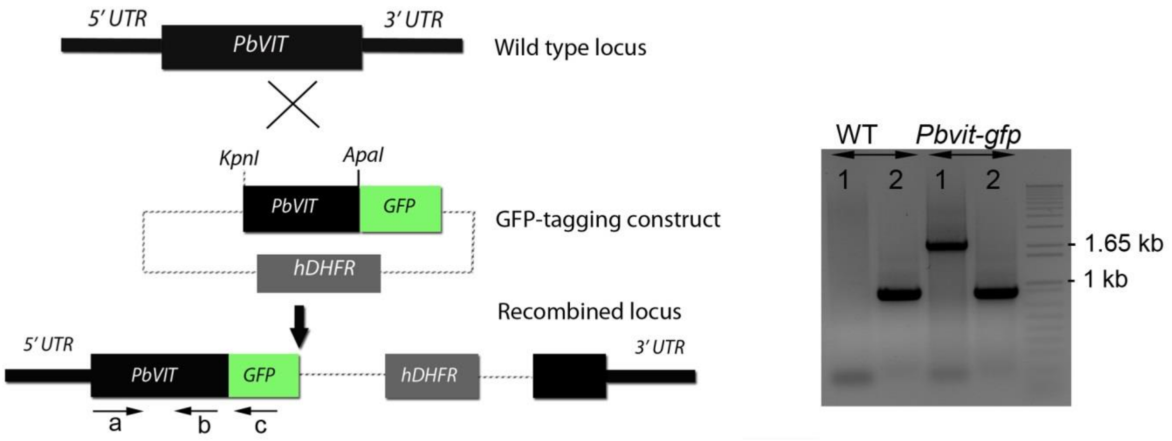

Supplementary Figure 4| Generation of *Pbvit-gfp* transgenic line. Single cross-over transfection strategy used to tag the endogenous PbVIT with a GFP sequence at the C-terminus (left) and genotyping of transgenic parasites by PCR (right). PCR lane 1 - integration detection (primers a+c, 2kb), lane 2 – control reaction (primers a+b, 0.8kb). Sequences of primers a, b and c are provided in Supplementary table 1 as following: a - PbVIT-gfp f, b – PbVIT-gfp r and c – GFPr.

Supplementary Figure 5

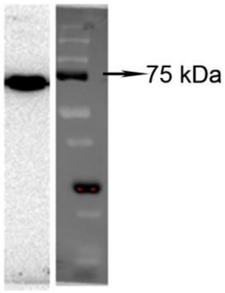

Supplementary Figure 5| Specificity of PbBiP antibody used as a marker for endoplasmic reticulum. The PbBiP antibody was designed to recognise a highly conserved C-terminal region of PBANKA_081890 (GANTPPPGDEDVDS) and was produced as a rabbit polyclonal antibody. Shown is a Western blot analysis of *P. berghei* blood-stage lysate, probed with 1:500 diluted PbBiP antibody and subsequently incubated with 1:5000 diluted HRP-conjugated secondary antibody. Blot was imaged with ChemiDoc XRS+ system. Predicted MW of PBANKA_081890 protein is 72 kDa.

Supplementary Figure 6

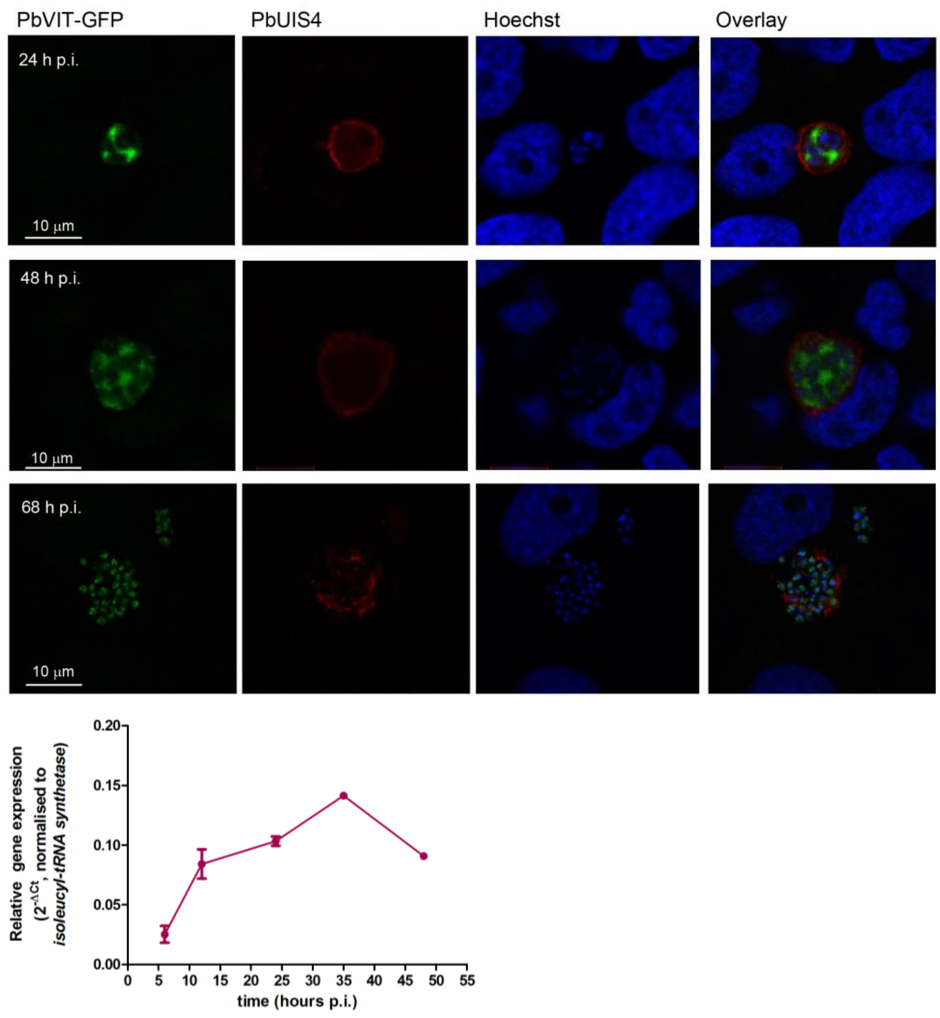

Supplementary Figure 6| Liver-stage expression of PbVIT. Protein and RNA expression of PbVIT was analysed over a time course of development in hepatoma cells *in vitro*. Top, hepatoma (Huh7) cells were infected with PbVIT-GFP sporozoites and immuno-stained 24h, 48 and 68h post-infection. Immuno-staining was performed with a rabbit polyclonal anti-GFP antibody (diluted 1:500; ab6556 from abcam) and goat PbUIS4 antibody (diluted 1:1000). Bottom, *Pbvit* RNA expression was analysed over a time course of development in hepatoma HepG2 cells infected with *P. berghei* sporozoites. For all time points *Pbvit* expression was normalised to the expression of *P. berghei* isoleucil-tRNA ligase. Shown are mean±S.E.M. values of 2 independent experiments.

Supplementary Figure 7

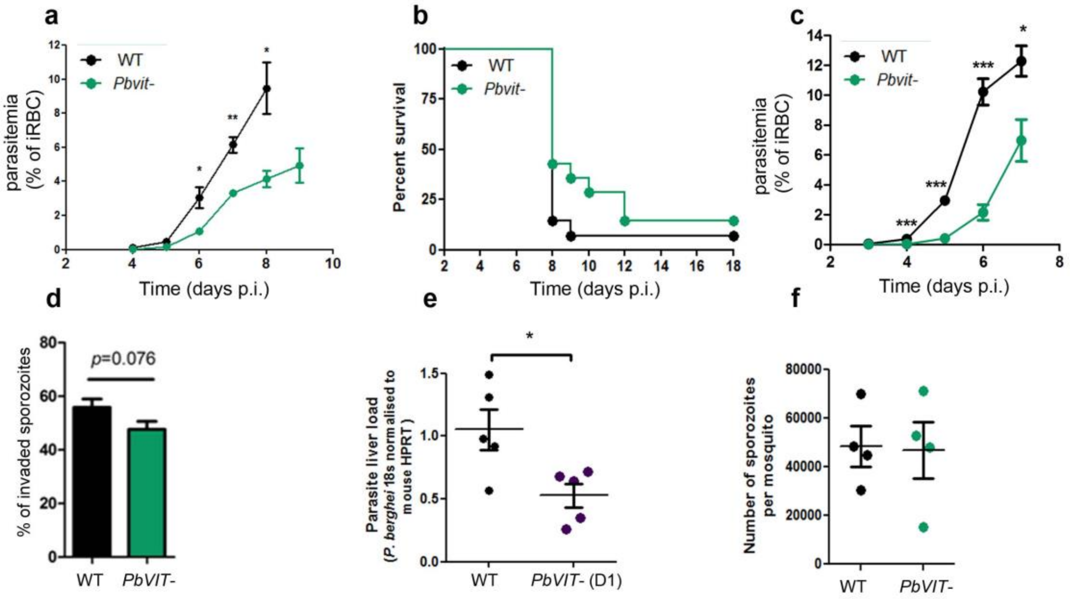

Supplementary Figure 7| Phenotype characterisation of *Pbvit-* . (**a**) Parasitemia of the C57Bl/6J mice following infection with 500 WT or *Pbvit-* (clone A2) sporozoites injected *i.v.* (N=5). Parasitemia of infected mice was determined by counting of infected RBC in Giemsa-stained blood smears. (**b**) Survival of C57Bl/6J mice infected with 500 WT and *Pbvit-* (clone A2) *P. berghei* sporozoites. Shown is a pool of 3 independent experiments, N=14 (p=0.19, Log-Rank Mantel-Cox test). (**c**) Full course of parasitemia during the infection of C57Bl/6J mice following infection (*i.v.*) with 10^4 wt *P. berghei* or *Pbvit-* iRBCs (clone A2), determined by counting of iRBC in Giemsa-stained blood smears (N = 5) (**d**) Efficiencies for WT and *Pbvit-* (clone A2) sporozoites to invade hepatoma HepG2 cells *in vitro* by comparing the number of invaded sporozoites 2h post infection to the total number of sporozoites, determined by immunofluorescence analyses of circumsporozoite protein as previously described [3] (shown result is a pool of 3 independent experiments, each performed with triplicates). (**e**) Analysis of the parasite liver load upon infection with a second, independent *Pbvit-* transgenic clone - D1, measured 45 hours post infection. Liver load was assessed by RT-PCR measurement of the parasite 18s RNA expression, normalised to mouse HPRT expression, shown are fold expressions relative to the average of controls- WT *P. berghei.* (**f**) Number of sporozoites derived from mosquitoes infected with WT and *Pbvit-* (clone A2) dissected 21 days post-infection. In (**a**), (**c**), (**d**) , (**e**) and (**f**) error bars denote S.E.M. and the asterisks denote significant differences using the two-tailed, unpaired Student's *t*-test: *P <0.05; ** P <0.01 and *** P <0.001.

Supplementary Figure 8

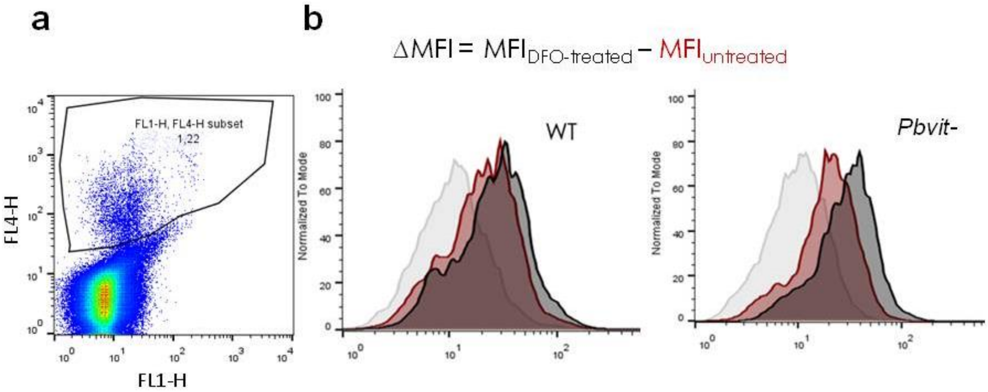

Supplementary Figure 8| Determination of labile iron pool of iRBC. (**a**) Gating strategy applied to select the *P. berghei*-infected RBC from non-infected RBC for further analysis by flow cytometry. FL4-H – Syto61 fluorescence (DNA stain); FL1-H – PhenGreen fluorescence (**b**) A representative histogram showing the PhenGreen fluorescence intensities of WT and Pbvit- of $FeSO_4$ – treated sample (light grey), non-treated (red) and DFO-treated sample (black). Labile iron pool is determined for each blood sample as ΔMFI (PhenGreen fluorescence of DFO-treated sample minus that of untreated sample), as previously described[4].

Supplementary Figure 9

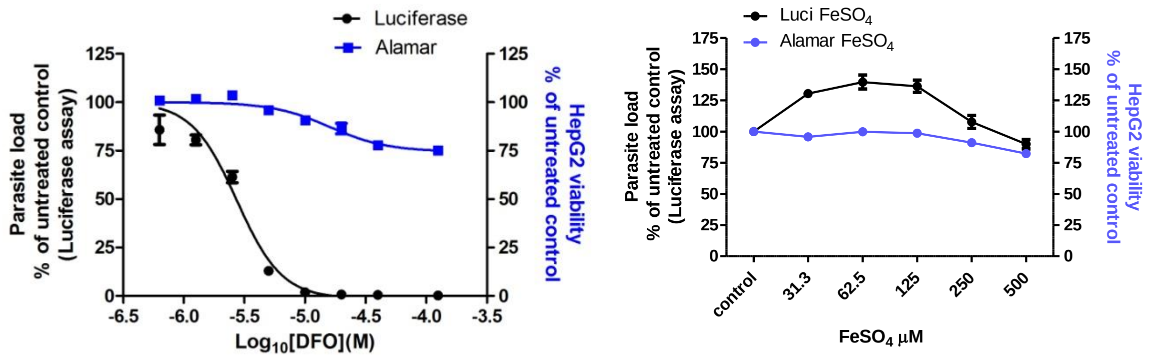

Supplementary Figure 9| Effect of DFO and $FeSO_4$ on *P. berghei* liver stage development *in vitro.*
Left, DFO EC_{50} determination for *P. berghei* liver stage development. Shown are means±S.E.M. of two independent experiments. Determined EC_{50} was 2.7 µM. Right, effect of $FeSO_4$ supplementation (in the presence of 0.5mM ascorbic acid) on *P. berghei* parasite load and HepG2 cell viability. Both for DFO and $FeSO_4$ experiments, HepG2 hepatoma cells were infected with *P. berghei* sporozoites expressing luciferase, and the parasite load was measured 45 hours post-infection by a luciferase assay, as previously described [5].

Supplementary Table 1. List of primers

**Generation of PbVIT knock-out construct**	
PbVIT-KO5'f	taGGTACCTATTTCTATATATGTTTCCG
PbVIT-KO5'r	TAGGGCCCTCTTTTTATTTTATTATTGCC
PbVIT-KO3'f	tcGATATCAGAGTTTAAGAATGTGGAATG
PbVIT-KO3'r	ATGCGGCCGCGTATAGTTACACAATACATG
**PbVIT knock-out genotyping primers**	
a	GCATTAATTCATAACTCTGATGTG
b	CAATGATTCATAAATAGTTGGACTTG
c	GATGTGTTATGTGATTAATTCATACAC
d	CCATGCCTTTAAATACATAATAATG
e	GTATGATACCTCTATTTTCTTATGTCC
**Generation of PbVITGFP construct**	
PbVIT-gfp f	taggtaccCATAGTCACTATCATAATCTCGATAAG
PbVIT-gfp r	tgggcccATCCCCTGAGTTTGTTTTAA
PbVIT SDMf	GATAGTAAGGATGCCAAAAATTTGGTAGAAATAAC
PbVIT SDMr	GTTATTTCTACCAAATTTTTGGCATCCTTACTATC
**PbVIT-GFP genotyping primers**	
GFPr	ACGCTGAACTTGTGGCCG
**Generation of yeast expression constructs**	
sPfVITf	aGGATCCAAAAATGCACAACTTGGATAAGGACAAC
sPfVITr	aTCTAGATTATTCGGAGATATTATTTTTCAAG
GPDprom_f	CTTCTGCTCTCTCTGATTTG
PUGr	CATTCAGGCTGCGCAACTG
optPfVITf	CTACTATAACGAAGATGTTGTATTGTC
optPfVITr	GAACCATACCAGCGATCATAC
**RT-PCR primers**	
PbVIT RTf	GTATGATACCTCTATTTTCTTATGTCC
PbVIT RTr	GTGGTAAACTGGGACTTGAATAAAC
Pb18s_f	AAGCATTAAATAAAGCGAATACATCCTTAC
Pb18s_r	GGAGATTGGTTTTGACGTTTATGTG
isoleuc.T-RNA ligase F (PBANKA_144000)	GCCATTCTGCTATTGTTCG
isoleuc.T-RNA ligase R	GCCGTTTTTTGGGTCATTTG
Mouse HPRTf	CATTATGCCGAGGATTTGGA
Mouse HPRTr	AATCCAGCAGGTCAGCAAAG

Supplementary References

[1] Solyakov, L. *et al.* Global kinomic and phospho-proteomic analyses of the human malaria parasite Plasmodium falciparum. *Nat Commun* **2**, 565, doi:ncomms1558 [pii] 10.1038/ncomms1558 (2011).

[2] Treeck, M., Sanders, J. L., Elias, J. E. & Boothroyd, J. C. The phosphoproteomes of Plasmodium falciparum and Toxoplasma gondii reveal unusual adaptations within and beyond the parasites' boundaries. *Cell Host Microbe* **10**, 410-419, doi:S1931-3128(11)00288-5 [pii] 10.1016/j.chom.2011.09.004 (2011).

[3] Sinnis, P., De La Vega, P., Coppi, A., Krzych, U. & Mota, M. M. Quantification of sporozoite invasion, migration, and development by microscopy and flow cytometry. *Methods Mol Biol* **923**, 385-400, doi:10.1007/978-1-62703-026-7_27 (2013).

[4] Clark, M., Fisher, N. C., Kasthuri, R. & Cerami Hand, C. Parasite maturation and host serum iron influence the labile iron pool of erythrocyte stage Plasmodium falciparum. *Br J Haematol* **161**, 262-269, doi:10.1111/bjh.12234 (2013).

[5] Ploemen, I. H. *et al.* Visualisation and quantitative analysis of the rodent malaria liver stage by real time imaging. *PLoS One* **4**, e7881, doi:10.1371/journal.pone.0007881 (2009).
